# Diabetic Foot Assessment and Care: Barriers and Facilitators in a Cross-Sectional Study in Bangalore, India

**DOI:** 10.3390/ijerph20115929

**Published:** 2023-05-23

**Authors:** Sudha B. G., Umadevi V., Joshi Manisha Shivaram, Pavan Belehalli, Shekar M. A., Chaluvanarayana H. C., Mohamed Yacin Sikkandar, Marcos Leal Brioschi

**Affiliations:** 1 Department of Computer Science and Engineering, B.M.S. College of Engineering, Bangalore 560019, India; 2 Department of Medical Electronics, B.M.S. College of Engineering, Bangalore 560019, India; 3 Department of Podiatry, Karnataka Institute of Endocrinology and Research, Bangalore 560019, India; 4 Medical Equipment Technology, College of Applied Medical Sciences, Majmaah University, Al Majmaah 11952, Saudi Arabia; 5 Medical Thermography Service, Neurology Department, Hospital das Clínicas, Sao Paulo University, Sao Paulo 01246-903, Brazil

**Keywords:** barriers, diabetic foot assessment, foot care practices, foot complications epidemiology, facilitators, foot care education, thermography

## Abstract

(1) Background: This cross-sectional study aims to highlight the assessment and foot care practices in an advanced clinical setting, the clinical characteristics of the patients, and to understand the barriers and facilitators for effective foot care from the perspectives of healthcare practices, resources, and patients’ socioeconomic and cultural practices, and other aspects in terms of new technologies for effective foot care such as infrared thermography. (2) Methods: Clinical test data from 158 diabetic patients and a questionnaire to assess the foot care education retention rate were collected at the Karnataka Institute of Endocrinology and Research (KIER) facility. (3) Results: Diabetic foot ulcers (DFUs) were found in 6% of the examined individuals. Male patients were more likely to have diabetes complications, with an odds ratio (OR) of 1.18 (CI = 0.49–2.84). Other diabetes problems raised the likelihood of DFUs by OR 5 (CI = 1.40–17.77). The constraints include socioeconomic position, employment conditions, religious customs, time and cost, and medication non-adherence. The attitude of podiatrists and nurses, diabetic foot education, and awareness protocols and amenities at the facility were all facilitators. (4) Conclusions: Most diabetic foot complications might be avoided with foot care education, regular foot assessments as the standard of treatment, and self-care as a preventive/therapeutic strategy.

## 1. Introduction

According to the International Diabetes Federation (IDF), India ranks second in the globe and first in Southeast Asia with around 74 million people with diabetes in 2021, accounting for one in every seven people worldwide [1]. Diabetes management costs around USD 114.4 per person per year in India [1]. The theme for World Diabetes Day 2021-23 is ‘Access to Diabetes Care’ [2], emphasizing the significance of diabetes treatment access.

In India, DFUs affect 15% of patients with diabetes during their lifetime. Evidence from the published literature showed 100,000 leg amputations/year due to diabetes-related problems and an expense of approximately USD 1960 for complete treatment of DFUs. In India, 25% of the diabetic population develop DFUs, of which 50% become infected, requiring hospitalization, while 20% need amputation. DFUs contribute to approximately 80% of all non-traumatic amputations in India annually. In addition, India is the most expensive country for DFU care, as 5.7 years (68.8 months) of an average patient’s income is required to pay for complete DFU therapy. In total, 50% of DFU patients who have one amputation suffer another amputation within the next 2 years [3]. The mortality rate following amputation [4,5,6] rises from 13–40% in 1 year to 39–80% in 5 years. This scenario necessitates a routine examination of the foot for the existence of any abnormalities. The problem is to properly adopt foot care while keeping costs in mind.

Diabetes-related foot care is one of the most ignored aspects of diabetes care in India. Due to social, religious, and economic compulsions, many people walk barefoot. Poverty and lack of education lead to the usage of inappropriate footwear and late presentation of foot lesions. [7]. Hence this study is an effort in closing the gap in knowledge of the barriers and facilitators specific to Indian population.

Foot treatments include diabetes-related foot education, therapeutic footwear, and routine foot care. Patients’ attention to foot care and self-management is the key to success among all the aspects that might aid in these duties. A combination of healthcare personnel and patient education, multidisciplinary foot ulcer treatment, prevention, and regular monitoring can lower amputation rates by 49–85% [8]. As a result, the IDF and WHO have set aims to cut amputation rates by up to 50% [9]. The International Working Group on the Diabetic Foot (IWGDF) recommendations [10] aided in the study design and methods.

This study was conducted at the Karnataka Institute of Endocrinology and Research (KIER, formerly known as the Karnataka Institute of Diabetology (KID)), an autonomous institute specialized in comprehensive diabetes care with a separate Department of Podiatry to deal with diabetes-related foot complications that is fully supported by the government in Bengaluru, Karnataka, India.

This study adds to prior research on diabetes-related foot complications [11,12,13], which focused on the clinical features of patients living with diabetes, personal attitudes, and habits, especially in industrialized nations.

Diabetes is common in older people usually, and hence, diabetes-related foot complications were more common in older persons with a longer history of diabetes, a higher BMI (Body Mass Index), hypertension, diabetes-related retinopathy, and smoking history. It was more common in men than in women, and it was more common in type-2 diabetes patients [11]. According to a study conducted in the rural districts of Udupi, Karnataka by Vibha et al. [12], advanced age, low social economic status, low literacy rate, unemployment, smokeless tobacco use, sedentary lifestyle, and longer duration of diabetes mellitus (DM) were all significantly associated with diabetic foot syndrome (DFS). It was also shown that “BMI, waist circumference, clinical parameters such as blood pressure, glycated hemoglobin, and presence of hypertension, hypercholesterolemia, and medication adherence, frequency of consulting physician, gender and religion” were not related to DFS. Another piece of research that is similar to ours is by Guell, Cornelia, and Nigel [13], who conducted an exploratory qualitative study in the Caribbean country of Barbados. According to the findings of the study, both healthcare practitioners and patients are more preoccupied with glycemic management, which has eclipsed the importance of foot care. The second obstacle noted by patients was opposition to new care responsibilities by healthcare providers, and the last barrier mentioned by patients was appointment/accessibility to podiatry services. Patient training sessions offered in all public care institutions for improving diabetes patients’ self-management were envisioned as possible facilitators.

A holistic unbiased view of regular foot assessment, clinical characteristics and socioeconomic status, foot care education retention rate of patients, and other aspects in terms of new technologies for effective foot care such as infrared thermography is reported here. The aim is to identify barriers and enablers to optimal diabetes-related foot care.

## 2. Materials and Methods

### 2.1. Research Design

This is a cross-sectional analytical study. The data of patients with diabetes were obtained at KIER and authorized by the Institutional Ethical Committee (Approval No: IEC-KIER/10/28.10.2017). The research was carried out over a year in 2019. All participants engaged in the study provided written informed consent. According to “Recommendations for the conduct, reporting, editing, and publication of scholarly work in medical journals” [14], the study was framed and organized for reporting.

### 2.2. Study Population

The study included 158 diabetic patients (103 men and 55 women) with DM.

Convenience non-probabilistic sampling was utilized in this study since it is part of another cohort study that involves the recording of thermal images of the foot to analyze and categorize the risk of diabetic foot problems [15,16]. Patients who came to the clinic for a routine foot exam, infection, discomfort, swelling, numbness, or wound dressing in the leg or DFU were chosen.

Sample size calculation was based according to Jyothylekshmy V et al. [17], where 49.45% of the study population had peripheral neuropathy, and 41.51% had non-healing ulcers. Diabetes is responsible for 40% to 72% of all lower-extremity amputations, with diabetic foot ulcer recurrence in 52% of the study group [18] and a 51% incidence of DFS [12]. As a result, we used 40% as the prevalence of at least one diabetic foot condition that leads to ulcer and hence amputation. Precision is considered to be one-fifth of the prevalence. As a result, the sample size for a 95% confidence level is around 145.

Adults over the age of 18 with type-1 or type-2 diabetes were eligible. Patients with diabetes who attend frequent foot evaluations, wound dressing, or follow-ups and who can provide informed permission demonstrating that they understand the study’s goal and methods were included.

Patients without diabetes attending the center for wound treatment or presently involved in another research study were excluded.

### 2.3. Clinical Foot Assessment and Foot Care Management Practices

**Figure 1:**
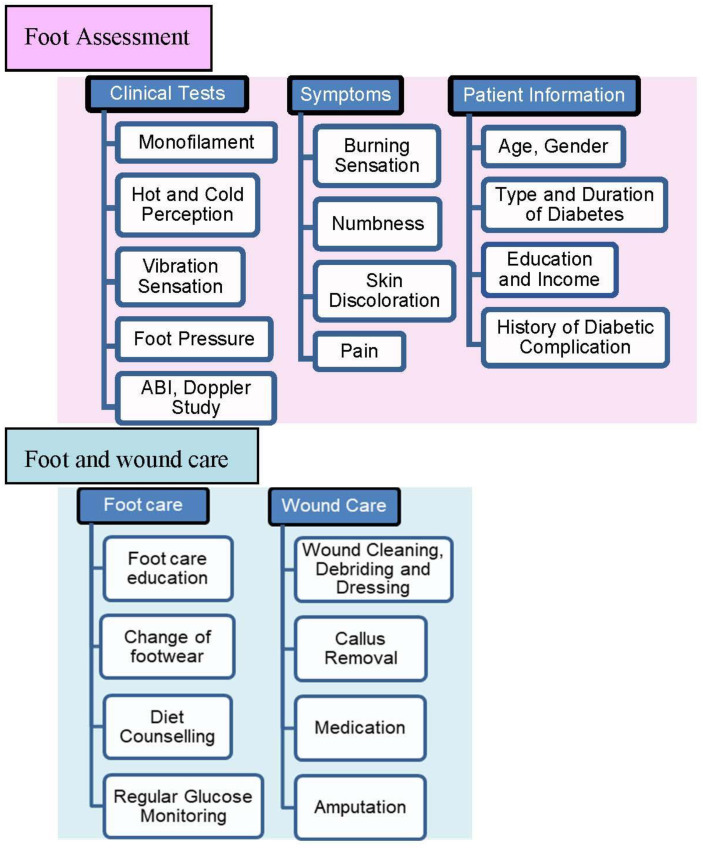
Diabetic foot assessment and wound management/care provided in the center.

The diabetes-related foot evaluation, information gathered, wound management, and treatment provided in the clinic are shown in Figure 1. This is accomplished by the diabetes-related foot examination and foot care management strategies recommended [19,20,21].

A typical foot evaluation regime in the center comprises the following: monofilament test (using 10 g filament), vibration perception test (VPT) (normal—<15 volts; grade I—16 to 25 volts; grade II—26 to 50 volts), hot perception test (HPT) and cold perception test (CPT) to identify the risk of small fiber neuropathy as shown in Table 1, pedography (to measure maximum peak pressure (MPP) and pressure distribution as an image), and vascular Doppler study.

To evaluate the sensory function of the foot in diabetes patients, we followed the IDF guidelines [10], which recommend at least 4 sites (first, third, and fifth metatarsal heads, and the hallux). In India, cracked heel and flat foot is common, leading to infections in these areas as well. Therefore, we have considered 6 sites (including medial longitudinal arch and the heel) for testing on each foot. The monofilament was applied perpendicular to the skin with enough pressure to bend it into a “C” shape. The patient was in a lying position with their eyes closed, and the examiner tested each site consecutively, alternating between the left and right foot. The results of the monofilament test were recorded in the patient’s medical records and used to guide treatment and management of the patient’s diabetes-related foot conditions.

The same 6 sites were considered for the VPT, HPT, and CPT tests. The study participants were lying down with their eyes closed during all these tests.

A vascular Doppler study is used to examine the risk of vascular ulcers using a portable Doppler. To identify PAD (peripheral arterial disease), the ABI (ankle brachial index) is automatically measured. Table 2 depicts the risk of vascular foot ulcers as found in the care center.

Following the foot examination, patients take the records to a podiatrist for interpretation and guidance based on the test results. In general, the patients were recommended to take drugs for neuropathy, exercise for pain, eat a healthy diet, and undergo additional testing such as an X-ray to rule out Charcot arthropathy and osteomyelitis. If an existing corn, callus, bruise, or wound is discovered, cleaning, debriding, corn and callus removal, and correct dressing are performed, and regular follow-ups are encouraged. Patients who come in for wound care would have these done on a daily or alternate-day basis. This is repeated until the wound heals/is treated surgically or is amputated in the event of non-healing wounds or spreading infection. Regardless of the presence of foot issues, podiatrists advise all patients to check their feet once a day for cracks, bruises, pricks, or sores.

### 2.4. Foot Care Education Retention Rate

In addition to the regular assessments, information on the foot care education retention is also collected for this study. Diabetes education and care influence diabetes care outcomes [22], and hence foot problems. As a result, the rate of foot care education retention was determined using a questionnaire (Table 3). The clinical practice guidelines on the diabetic foot provided by the IDF in [20] were used to frame these questions. If the patient answers ‘yes’ to a question, he or she receives a score of one. A score of 1 indicates that the patient replied ‘yes’ to one of the ten questions presented.

## 3. Results

### 3.1. Baseline Characteristics of Patients

Table 4 shows the baseline characteristics of patients who attend the facility.

The male preponderance seen in this investigation was consistent with prior studies [22,23]. Patients with type-2 diabetes outnumbered those with type-1 diabetes, implying that type-2 diabetes patients are more vulnerable to diabetes-related foot problems. These findings are consistent with worldwide findings [11].

**Figure 2:**
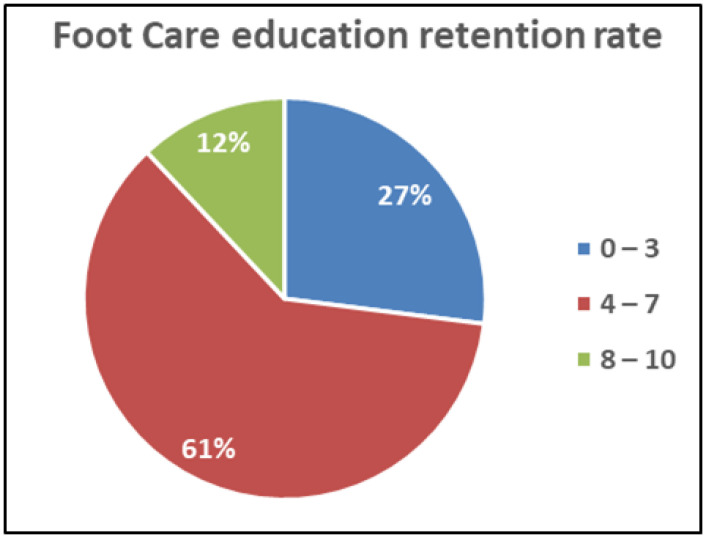
Foot care education retention rate among the subjects.

Numbness is seen in around 50% of patients, according to Table 4. DPN was verified by VPT and HPT, and the frequency of DPN in our sample of 43% is consistent with earlier investigations in India [24]. Based on the questionnaire in Table 3, the foot care education retention rate indicated in Figure 2 was determined. The most favorable responses were obtained for questions 1, 2, 9, and 10.

Only 30.5% of patients acknowledged receiving foot care instruction. This is low because a group of skilled podiatric nurses provided foot care instruction to all patients registered with the hospital.

### 3.2. Statistical Test Results

To see if amputations and foot care education retention rates are related, we ran a chi-square independence test on the data. The statistical test result indicates that the statistical test is significant (*p*-value < 0.05), and so, amputations are dependent on the retention rate of foot care instruction (at 99% confidence level).

Males have a higher risk of diabetes problems than females. Male patients had a greater risk of diabetes complications than female patients, with an odds ratio of 1.18 (CI = 0.49–2.84). This is supported by several additional investigations [11].

Males have a greater training retention rate than females. Male patients appear to recall lower-level foot care instruction more than female patients, with an odds ratio of 0.89 (CI = 0.40–1.96).

The presence of prior diabetes problems enhanced the likelihood of DFUs with OR 5 (CI = 1.40–17.77).

### 3.3. Risk Classification of Patients

The International Diabetes Federation (IDF) Z-card or the diabetic foot screening pocket chart [25], presented in Table 5, provides standards for health professionals to identify, assess, and treat diabetes-related foot patients earlier in the “window of presentation” between when neuropathy is diagnosed and prior to developing an ulcer.

**Figure 3:**
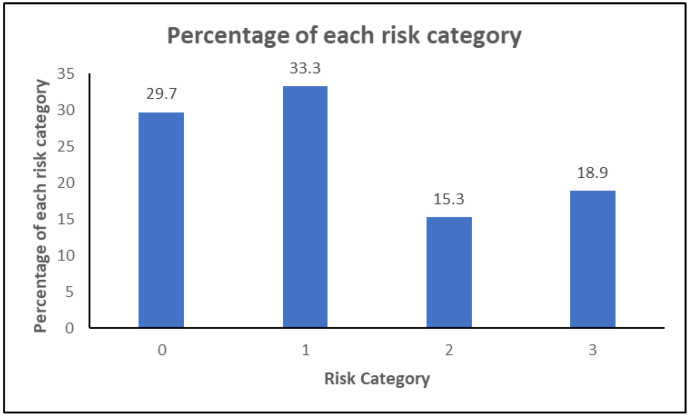
Risk patient classification of study participants as per the IDF Z-card.

The risk classification of our research participants is shown in Figure 3. This is based on the guidelines in the diabetic foot screening pocket chart [25] and the extract from the pocket chart Z-card.

Figure 3 shows that the percentage of patients at low and moderate risk is higher than the percentage of patients at high and very high risk. This demonstrates that once a risk is identified early on, it is possible to control the progression to high and very high risk. Nonetheless, the proportion of very-high-risk patients is slightly higher than the proportion of high-risk patients. This is because of the fact that once it enters risk category 3, the chances of controlling further complications are slim because it is at a very advanced stage.

### 3.4. Barriers and Facilitators

**Figure 4:**
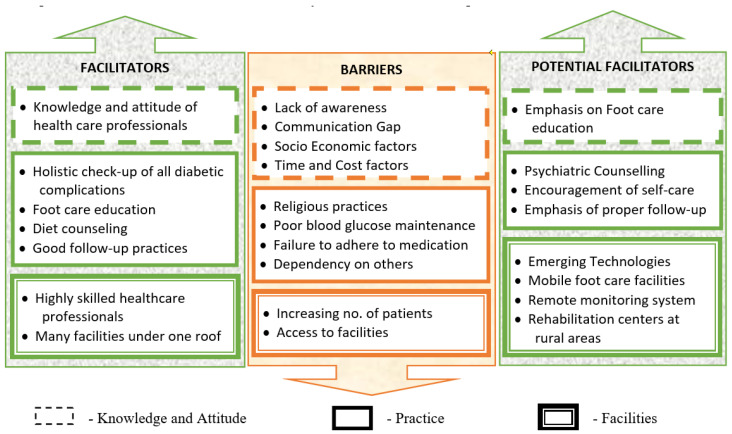
Facilitators, barriers and potential facilitators.

Despite all examinations and wound care, there are still impediments to good foot care that are comparable to those documented in the literature [26,27,28,29,30]. The hurdles and facilitators particular to diabetes-related foot care as recognized by health professionals [28] are also applicable to this center (See Figure 4), and there are issues in terms of public health and health systems for diabetes treatment and management [29]. Another study [20] showed that patient knowledge and attitudes, self-care, and socioeconomic level all play important roles in good foot care as assessed by multidisciplinary healthcare providers. However, this is skewed because it only included the opinions of healthcare professionals.

#### 3.4.1. Barriers

##### Lack of Awareness

Patients who visit the foot care facility for the first time are unaware of the diabetes-related foot issues that might emerge and the associated difficulties. The importance of foot care and frequent foot examination is often overlooked. This is confirmed by the research of Soumya et al. and Saurabh et al. [31,32]. People aren’t aware that ill-fitting shoes, harsh bottoms, and going barefoot can lead to diabetes-related foot issues. Patients ignore foot examinations and only glance at their feet when there is blood on the floor, or the cut becomes painful. This is confirmed by a statistical test that shows that persons with diabetes for a longer period retain more foot care knowledge.

##### Religious Practices

Men and women throughout most of the country go barefoot to local sites and temples, as is customary with any religious activity, as recorded in Vibha et al. [12] and Guell et al. [13]. Few men and women, particularly in South India, do not wear footwear even for a month when on pilgrimage to specific temples. They consider it a religious activity. Fasting during Ramzan (a religious festival) contributes to poor diabetes control and, as a result, poor diabetes-related foot outcomes.

##### Time and Cost Factor

Most patients arrive early in the morning to perform fasting glucose testing, post-prandial sugar tests, diet counseling, ECG checks, and then meet with a diabetes-related expert if necessary. They may also be requested to perform eye examinations and foot assessment tests. It takes two days to conduct all of the tests and meet the podiatrist. The tests for foot examination take at least 30 min for each patient. The IWGDF [10] advises foot evaluation at least once a year for diabetes patients without current difficulties and once every 6 months or 3 months for individuals at risk, depending on the risk or complication involved. Patients going from adjacent communities must factor in travel time and expense, which is a significant obstacle to efficient wound treatment.

##### Socio-Economic Factor

People in the educated middle and upper middle classes (which account for only 12% of our research group) had higher levels of awareness and foot care knowledge. When a patient’s financial situation is bad, individuals begin to ignore foot issues due to the lack of financial assistance. People are also less alert when they live in places where there is a dearth of understanding about the complications and how to prevent them. This is similar to what Agha et al. discovered in [26].

##### Working Environment Factor

**Figure 5:**
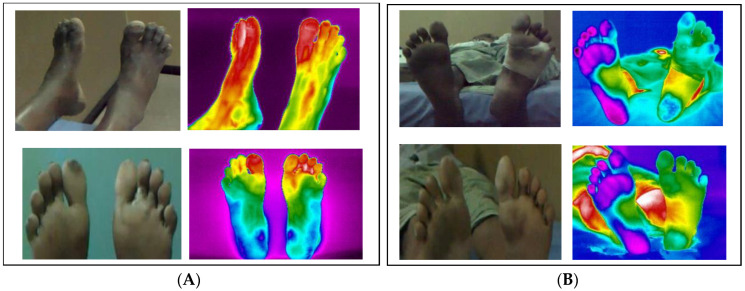
(**A**) A patient’s foot from a construction site with a blister and (**B**) another patient’s foot from a housekeeping occupation with a web space ulcer.

Diabetes-related foot sufferers have additional problems in the workplace. For example, a man working in the construction business had blisters on his foot as a result of contact with cement and sharp things such as steel, while another man developed web space infection as a result of continual soaking of his foot due to housekeeping activities. Figure 5 depicts the visual and infrared thermal images that were captured.

##### Access to Specialized Foot Care and Increasing Number of Patients

In Bangalore, there are three specialized foot care facilities where the same type of test is performed. There are no specialized centers in adjacent areas, thus individuals from a 100 km radius in Karnataka come to this facility. The facility serves patients not just from Karnataka but also from neighboring states including Andhra Pradesh and Tamil Nadu. The number of patients continues to rise, putting a strain on podiatrists. When an operation to amputate a foot or toe is scheduled, patient waiting times increase, forcing the facility to limit the number of new patients enrolled. This is a major concern for patients who travel significant distances (approximately 100 km from Hindupur in Andhra Pradesh and Krishnagiri in Tamil Nadu) to see the podiatrist.

##### Dependency by the Patient

Patients with diabetes, particularly women, expect someone to accompany them to the facility. Owing to this dependency, the visit gets delayed, and hence, the wound becomes infected. This further causes delay in the therapeutic procedure. Similarly, patients with limited mobility also expect a member of their family to accompany them to the care facility. Such dependencies postpone effective treatment at the appropriate moment, resulting in poor wound care results. Eight patients had arrived in pairs. Three of the pairings were husband and wife, while the fourth was a mother and son.

##### Communication Gap

In total, 75% of patients see both a diabetes specialist and a foot care specialist and receive medical prescriptions from both. When the foot care professional instructs the patient to discontinue the drug after a certain length of use, some individuals misinterpret it for the complete set of prescriptions and stop taking all medications, including diabetes medicines. This is a serious issue since it raises blood glucose levels, causing nerve damage. Certain people quit taking their drugs abruptly before the time limit because they have produced some undesired side effects without informing their doctor. This, once again, impedes successful therapy. Few people do not finish their antibiotic course, and as a result, they develop resistance to some of the antibiotics, requiring the doctor to provide a larger dose of another potent and expensive antibiotic to combat the infection.

##### Poor Blood Glucose Level Monitoring

Many patients only check their blood glucose levels once a year, and others go even longer periods. Diabetes management failure results in poor diabetes-related foot care outcomes [28]. Despite the fact that the aforementioned hurdles impede good foot care treatment and wound management, some facilitators attempt to enhance foot care wound management at the center, as shown in the next section.

#### 3.4.2. Facilitators

The presence of highly experienced surgeons and nurses for quick action—limb salvage surgery and vascular surgery—are facilitators, as is assessment of various diabetes problems such as nephropathy, retinopathy, and cardiology under one roof. Self-foot care and patient education, as documented in [30,31,32], are also major facilitators here, resulting in a decreased proportion of high-risk patients. Diet counseling to keep blood glucose levels under control, physiotherapy to alleviate discomfort and promote mobility, provision for purchasing specialist diabetes-related footwear within the facility, and the surgeons’ attitude and degree of care and concern for patients are also facilitators.

#### 3.4.3. Potential Facilitators

Emerging technologies [33] such as laser Doppler flowmetry, elastography, infrared thermography, plantar pressure, and pressure gradient system for DFUs, as well as other rehabilitation modalities such as off-loading devices and electrotherapy, might be useful. Also available are foot care education experts in addition to podiatric doctors to give information and instruction to patients. Furthermore, as indicated in [34], health education on diabetes by school instructors would have a significant influence on the awareness of diabetes as a disease, as well as its prevention and treatment, which would enhance the result of diabetes-related complications. During a pandemic, digital/virtual diabetes clinics [35] potentially enhance diabetes-related foot care outcomes.

Monitoring self-care activities by delivering automated SMS reminders to examine the foot regularly will also be helpful. Healthcare on the go, such as mHealth [36], might be investigated because it has been demonstrated to be possible and acceptable. Identifying patients who might serve as foot care education specialists would improve diabetes-related foot outcomes [37,38]. The authors reported using a non-invasive and non-contact screening technology, such as an infrared thermal image-based diagnostic system, as part of their larger investigation [15,16]. Infrared thermography and visual systems are used in remote monitoring systems to save time and travel, as documented in [39]. Rural foot care camps are being organized. Rehabilitation and mental therapy are provided to impacted individuals. In addition, new specialist foot care clinics with suitable resources could be established in surrounding rural regions.

### 3.5. Strength and Limitations of the Study

This is the first population-based research on diabetes-related foot care practices in metropolitan Bengaluru, conducted in partnership with the center by an external contributor. It discusses foot examination and management strategies, as well as the prevalence of diabetes-related foot and associated problems. The study also examines the challenges and enablers of good foot care. The findings of this study indicate that excellent practices and foot care education lower the occurrence of diabetes-related foot problems.

A high-sample-size investigation is required for more exact results. Recent global developments warrant a targeted investigation on the effect of the COVID-19 pandemic on foot care practices in order to identify further gaps and requirements that might bring more value to this research. Surprisingly, the influence of COVID-19 on the treatment and care of diabetes and its consequences described by Raman et al. [40] likewise shows comparable obstacles to those presented here.

Although our study provides valuable insights into the topic, caution should be taken when applying the findings to other populations or settings. Further research is needed to confirm the results and explore potential differences across populations.

## 4. Conclusions

According to the algorithm for “prevention of diabetes mellitus and diabetes-related foot” [41,42], our study identifies as barriers a lack of awareness, neglect of self-care and socioeconomic status, limited resources to cater to an increasing number of patients, and difficulty accessing facilities from rural areas. The presence of highly skilled surgeons and nurses with the correct mindset, as well as the center’s amenities under one roof, proved to be the most effective and supporting facilitators. An important finding from this study is that, even 20 years after Viswanathan’s [38] study, the necessity and implementation of foot care must be reaffirmed and improved to ensure a reduction in risk of deterioration. Significantly, the retention rate of foot care education is lower, which is a highly hopeful feature that underlines the importance of improving foot care education and monitoring to minimize the prevalence of DFUs. Patients who took diabetic foot education seriously had a decreased risk of lower-limb amputation evident from the statistical test results.

DPN, corns or calluses, previously treated foot ulcers, diabetic retinopathy, cardiovascular disease, and diabetic nephropathy are among the numerous consequences. This is consistent with research that found a strong association [24] between knowledge score and gender, diabetes duration, employment, level of education, place of residence, having DFU, hospital stay history, and amputation history.

Our analysis found 18.9% of the participants to be part of the very-high-risk group. This shows that the institute is quite good at addressing ‘at-risk’ patients such that they do not develop diabetic foot ulcers (DFU is 6%). Alternative means of assessment and remote monitoring, which need patient involvement, might be viewed as a way forward to avoid/bypass some of the hurdles stated. This study also asks for a government push to establish additional such institutes in distant locations, as well as to hold mobile awareness camps to highlight the underappreciated importance of foot care.

## Figures and Tables

**Table 1:** Heat perception test used to classify the risk of small fiber neuropathy (°C).

Condition	Perception	Temperature
Normal	Hot Cold	<42.4 °C >19 °C
Mild	Hot Cold	42.5–45.4 °C 15–19 °C
Moderate	Hot Cold	45.5–48 °C 11–15 °C
Severe	Hot Cold	>48 °C <10 °C

**Table 2:** Classification of risk of vascular foot ulcer (ankle brachial index—ABI).

Condition	ABI	Risk Classification
Normal	0.9–1.2	Risk is small
Definite vascular disease	0.6–0.9	Risk is moderate and depends on other risk factors
Severe vascular disease	Less than 0.6	Risk is very high

**Table 3:** Questionnaire for calculating foot care education retention.

Q.No.	Question
1.	Do you inspect your foot regularly?
2.	Do you wear footwear regularly while walking?
3.	Do you wash your feet regularly with warm water?
4.	Do you keep your foot dry?
5.	Do you wear special soft shoes?
6.	Do you apply moisturizer?
7.	Do you go for regular foot monitoring?
8.	Do you regularly check your blood sugar levels?
9.	Do you cut your nails regularly?
10.	Do you report the presence of blisters/corns to a foot specialist?

**Table 4:** Baseline and diabetes-related foot characteristics.

Baseline Characteristics	All Subjects (Mean ± Standard Deviation)	
Age (years)	58 ± 12	
Gender		
Female	55	
Male	103	
Diabetes duration (year) Type 1 DM Type 2 DM	11 ± 7 27 131	
Education		
Diploma or no degree	122	
University degree	36	
Job profile		
Low	57	
Medium	37	
High	12	
No job	52	
**Diabetic foot characteristics**	**Present (in %)**	**Absent (in %)**
Burning	50	50
Numbness Diabetic peripheral neuropathy (DPN) Heredity	49 43 48	51 57 52
Trauma	5	95
Deformity	13	87
Foot care training	60	40
History of/leading to amputation	2	98
Special footwear	18	82
Previous other diabetes-related complications	35	65
Diabetic foot ulcer	6	94

**Table 5:** Risk classification (extracted) from IDF Z-card.

Risk Category	0	1	2	3
**Assessment**	Normal Plantar Sensation	Loss of Protective Sensation (LOPS)	LOPS with either High Pressure or Poor Circulation (Peripheral Arterial Disease) or Structural Foot Deformities or Onychomycosis	History of Ulceration, Amputation or Neuropathic Fracture
**Risk Classification**	Low	Moderate	High	Very High

## Data Availability

The datasets created or analyzed at the Karnataka Institute of Endocrinology and Research (KIER) for the current study are not publicly available because they contain information that could jeopardize ongoing research and compromise participants’ privacy or consent, but are available from the corresponding author on reasonable request.
